# The times, they are a‐changin'

**DOI:** 10.1111/ppl.13716

**Published:** 2022-06-14

**Authors:** Sam W. van Es

**Affiliations:** ^1^ Department of Plant Physiology Umeå University Umeå Sweden

**Human induced climate change is, due to an enormous lack of resolve from governing bodies and humanity as a whole, a thing we sadly have to learn to live with. In the 2015 Paris agreement, the world decided to cut emissions and pursue efforts to limit the temperature increase to 1.5°C above preindustrial levels. However, in the latest IPCC report it became clear that not enough is being done to keep that limit within sight. This will have serious consequences for the climate on our planet, with possible devastating effects on ecosystems, their species composition, distributions and biodiversity. The precise effect a changing climate will have on a specific ecosystem is challenging to predict. In an effort to gain insight into this, Aspinwall et al. characterized several traits of four species of plant dominant in a subtropical forest, when subjected to increasing temperatures.**

The effect of rising temperatures on the climate is hard to predict accurately, but the general prognosis is that the average global surface temperature will rise, that this rising temperature will be unevenly distributed over the planet, precipitation patterns will change, and more extreme weather events will occur (Paris agreement, 2015; IPCC, 2022). Though hard to anticipate, it is suggested that this will result in the moving of climatic zones, for example, shifting subtropical regions to a tropical climate, or the Mediterranean climate shifting toward a more desert‐like climate (Mahlstein et al., 2013). It goes without saying that this will have tremendous consequences for the people, flora, and fauna living in those regions.

The effects of a changing climate are already being felt by winemakers in Southern Europe. In Spain and Italy, farmers are moving to higher altitudes and planting grapevine on north‐facing slopes to mitigate the effects of a changing climate (Hannah et al., 2013). And what would have been absolutely unthinkable 50 years ago is slowly becoming a reality, wine from Scandinavia. Higher temperatures make for a longer growing season in summer and winters become milder, allowing the cultivation of a previously exotic crop. Grapevine (*Vitis vinifera*) is an economically important crop species, motivating people to move them to climate zones where they can thrive. This of course does not apply to the average tree, shrub, or herb and it is therefore thought that changing climate will change the species composition in certain areas over time.

**FIGURE 1:**
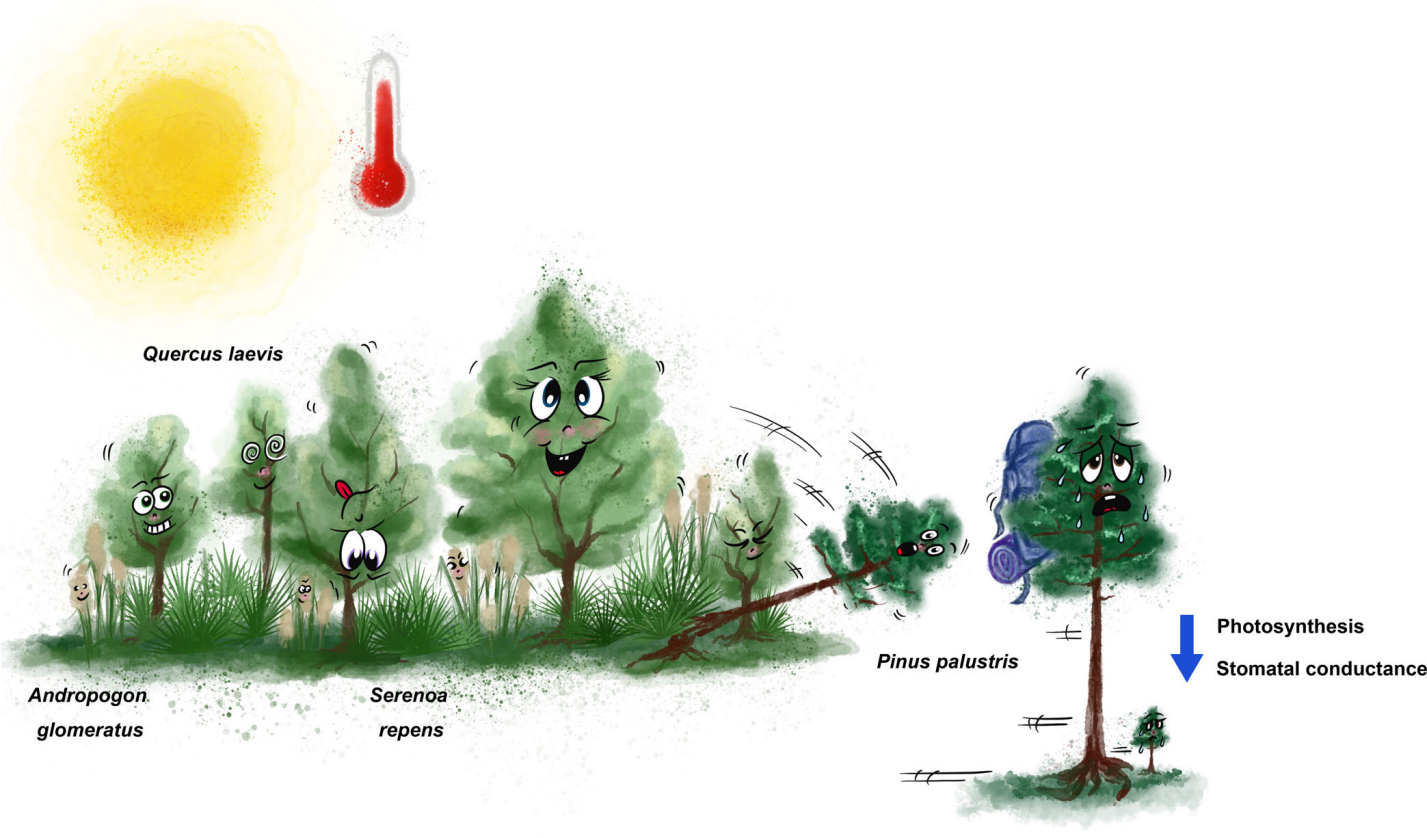
**The effect of rising temperature on species composition**. *Pinus palustris*, a needled evergreen, might succumb to the heat through a reduction of its photosynthetic efficiency and stomatal conductance. At the same time, the other species used in this study ‐ *Serenoa repens* (palm), *Andropogon glomeratus* (C4 grass) and *Quercus laevis* (broadleaved deciduous tree) ‐ seemed to be capable of handling the rise in temperature.

Plant species generally have a range of conditions in which they prosper. The European oak (*Quercus robur*) for example grows all over Europe, from southern Scandinavia to Turkey, and from Ireland to well within Russia (Caudullo et al., 2017). Plants growing on the limit of their distribution will likely suffer first from a change in climate, and the southern limit might become too hot and dry for the European oak. The article of Aspinwall et al. (2022) in the current issue of Physiologia Plantarum, aims to investigate the effect of a rise in ambient temperature on four plant species, common and dominant in a subtropical forest (FL, USA). The authors found that two monocot species distributed in warmer climatic regions, a palm, and a grass, were able to handle the rise in temperature. However, the species that is growing close to its warmest geographic or physiological limit, the evergreen Longleaf pine *Pinus palustris*, was suffering from the rise in temperature. Its seedlings are expected to suffer even more as they are more susceptible to a rise in temperature than the full‐grown specimens of this species. This might contribute to the complete disappearance of this species from this geographical area in the future, if indeed the subtropical forest turns into a tropical one (Figure 1). Time will tell what precise effect this has on the forest in Florida, but it is generally thought that loss of biodiversity through the removal of a key species in an ecosystem has a profound negative effect on the biomass production of that ecosystem (Flombaum & Sala, 2008).

The response to a changing climate will depend on many different factors in addition to the rise in temperature, such as changes in precipitation, general weather patterns, and the resilience of the species in question. We might conclude therefore that yes, the times are changing toward an uncertain future, but studies such as the one highlighted in this spotlight are essential in providing us with some insight as to where we are headed.
